# Walking out of the shadows: Exploring the complexities of motherhood and intergenerational realities in the families of three Taiwanese comfort women survivors

**DOI:** 10.1177/13634615251342640

**Published:** 2025-06-30

**Authors:** Shu-Hua Kang, Myriam Denov

**Affiliations:** School of Social Work, 5620 McGill University

**Keywords:** comfort women, conflict-related sexual violence, intergenerational trauma and resilience, motherhood, Taiwan

## Abstract

Previous studies have found that Taiwanese comfort women survivors faced multiple forms of trauma from the comfort women system, and that societal prejudice against women's sexual victimization further impacted their marriages. However, there is minimal research exploring how sexual trauma may have impacted comfort women survivors’ experiences of motherhood, alongside the consequences of survivors’ experiences on subsequent generations. This article explores the perceptions of eight family members, including the second and third generations, of three deceased Taiwanese Han-Chinese ethnicity comfort women survivors. In particular, we trace family members’ perspectives of survivors’ mothering, and how family members were impacted by their mother's experiences as a comfort woman. To ensure participants’ anonymity, findings are presented using composite narratives. The narratives illustrate the ways in which survivors’ sexual trauma reportedly impacted survivors’ family formation and mothering, and had long-term effects on survivors’ offspring. According to family members, ambivalent mother–daughter relationships and conflictual relationships resulting from the preferential treatment of the male offspring were found. In addition, family members’ psychological well-being, marriages, personality, and parenting were impacted by survivors’ sexual trauma and conflictual family dynamics. Nevertheless, participants showed great strength and capacity under challenging circumstances and actively undertook their healing journey. We highlight the importance of providing culture-driven multilayered services for the families of comfort women survivors to foster intergenerational resilience, enabling them to continue to “walk out of the shadows” of conflict-related sexual violence.

## Introduction

Conflict-related sexual violence (CRSV) is one of the most recurring forms of wartime violence (Alison, 2007) that significantly impacts all genders, but particularly women and girls (United Nations, 2021). Among the numerous incidents of CRSV, the sexual slavery system during World War II (commonly known as the “comfort women” system), employed and operated by the Imperial Japanese government and military, offers an extreme example of institutionalized sexual violence against women (Watanabe, 1995). It has been estimated that between 50,000 and 200,000 women across Asia were victimized between 1932 and 1945 (Yoshimi, 2000). These women were raped by Japanese soldiers under strict surveillance and experienced multiple and severe forms of violence. Many of these women suffered profound mental distress, with many later dying from illness or suicide (Yoshimi, 2000). Moreover, with Japan's defeat in 1945, large numbers of comfort women were killed by the Japanese military to destroy evidence of the comfort women system (Qiu et al., 2013).

Among the comfort women victims, at least 1,000 women were of Taiwanese origin (K.-S. Lee, 1999). After decades of silence and silencing, through the support of the transnational redress movement for the victims of Japanese military sexual slavery of the comfort women system, since 1992, 59 Taiwanese survivors have been identified by historians and activists, including 47 Han-Chinese and 12 Indigenous peoples victimized in Taiwan and overseas. Studies show that after the war, these comfort women survivors faced additional trauma from societal prejudice regarding women's sexual victimization that impacted survivors’ marriageability, and their status as wives and mothers, as defined by traditional gender norms for a woman's life path (Chiang, 1996; Taipei Women’s Rescue Foundation [TWRF], 1999). Many survivors faced reproductive health issues from their sexual trauma, often resulting in infertility, which caused tremendous loss and shame in a Confucianism society that valued women's reproductivity for family succession (Chiang, 1996). Furthermore, 29% of survivors became pregnant or gave birth to children as a result of repeated rape and sexual slavery within the comfort women system (Chu, 2009). Many survivors, including Indigenous survivors, were often rejected by their communities because of their sexual victimization (Kang & Wang, 2025; TWRF, 1999).

Researchers on Korean comfort women survivors have addressed the lifelong psychological distress caused by comfort women's experiences, such as the high prevalence of post-traumatic stress disorder (PTSD) among survivors (J. Lee et al., 2018; S.K. Min et al., 2011) and the long-term impact of sexual victimization on comfort women survivors’ life course (Park et al., 2016). Notably, the authors of one study highlighted the intergenerational transmission of comfort women's sexual trauma to their offspring in the form of psychiatric disorder symptoms among comfort women survivors’ second generation (J. Lee et al., 2019). Although it has been argued that comfort women are victims of multiple structural oppressions involving their race, class, and gender (P.G. Min, 2003; Soh, 2008; H. Yang, 1997), little in-depth attention has been paid to survivors’ experiences of motherhood, and also, the ways in which the families including second and third generations of comfort women survivors may have been impacted by their mother's history of wartime sexual violence and sexual slavery.

To address this gap, we drew upon qualitative data from the families of three deceased Taiwanese Han-Chinese ethnicity comfort women survivors, including from the second and third generations. First, we provide contextual information on the socio-historical, cultural, and familial structures embedded in these three Taiwanese comfort women's families to situate the readers. Next, we explore how complex structural oppression may have shaped the deceased survivors’ experiences of motherhood and the intergenerational realities experienced by their children and grandchildren. We sought to answer the following questions from family members: How did survivors’ sexual trauma interplay with structural factors shaping motherhood and parenting? And how did survivors’ experiences of multiple forms of violence impact subsequent generations?

## Literature review

### Structural realities, gendered norms, and family formation: The impact on Taiwanese comfort women and their descendants

Traditional Han-Chinese gendered norms have been highly influenced by Confucianism, a system of sociopolitical order, ethical doctrine, and a religious tradition, that originated in ancient China and influenced many East Asia countries as guiding principles of people's behaviors and social culture (Yao, 2000). Confucianism has been essential to shaping cultural constructions of family formation in Taiwan, thus shaping the realities and experiences of comfort women survivors and their families, particularly the importance of continuing patrilineal family lines. Male descendants are considered important and preferred in order to carry on the family line (L.-S. Yang, 1995). In addition, until the 20th century, a woman was considered a “temporary” member of her biological family. When a woman married, her formal tie with her birth family was severed (M. Wolf, 1972). Unmarried women were excluded from their birth family line. It was only through marriage that a woman was deemed eligible to have a status in her husband's family line, and, importantly, could be worshipped by her male descendants after her death. The importance and significance of a woman getting married and having a male descendant was thus paramount to a woman's survival, status, and well-being in this cultural context (Chang, 2000; H. Yang, 1995). Traditionally, it was acceptable to divorce a wife who failed to provide a male offspring (Chen, 2006). It was only when a married woman gave birth to a child that she could justify her rights in the family (M. Wolf, 1972). These traditional gendered norms highlight the “expected” path for a Taiwanese woman. Since the 1970s, the feminist movement emerged in Taiwan and prompted many awareness campaigns and legal reforms to emancipate women from these patriarchal structures—campaigns that continue today.

Most of the Han-Chinese comfort women survivors identified by historians and activists came from impoverished families, which prompted them to enter the labor market at a young age, work in entry-level jobs, and become targets of trafficking into the comfort women system (Chu, 2009), highlighting how class oppression was embedded in their lives. These survivors were born and raised during the Japanese colonial era (1895–1945), and most married after World War II (Chu, 2009). Although there is a scarcity of research addressing Han-Chinese survivors’ experiences of motherhood and family life, Chiang's (1996) study of 33 Han-Chinese comfort women survivors shows the influence of traditional gender norms and their sexual victimization on survivors’ marriages and decisions to adopt children. Chiang notes the intense shame of these women due to their sexual victimization and societal perspectives on women's sexuality. Their sense of self-worthlessness led to pessimism about their marriageability, an expected path for women and an essential means for them to gain economic security, particularly at that time. In addition, 60% of the women in Chiang's study were infertile, likely because of repeated rape. In response, many women chose to adopt children from their relatives or acquaintances in order to pass on family legacies, to ensure their economic security in the future, and to have offspring to worship them after their death.

It should be noted that 53% of Han-Chinese comfort women had been, as children, adopted out and raised to provide free labor or become future brides (Chu, 2009), reflecting the patriarchal oppression omnipresent in these women's lives. The unique customs related to adopted daughters (*yang-nü*) and daughters-in-law (*tong-yang-x*i) were prevalent in Taiwan in the 19th and early to the mid-20th centuries. Usually, girls were adopted into a family, to guarantee a wife for sons in adoptive households, or they served as laborers. If adoptive parents had no male child, adopted girls would be required to have an uxorilocal marriage (*ru-zhui*), a man would be married into his wife's family, for patrilineal succession (Chang, 2000; A. P. Wolf & Huang, 1980). Usually, the husband assumed at least partial duties of a son and supported his wife's parents. It was typically agreed that the first child must take the maternal surname (Li et al., 2020; A. P. Wolf & Huang, 1980). Chen (2021) suggests that these adopted daughters/daughter-in-law girls lacked care and nurturing from their own biological mothers, which may have led to a complicated perception and meaning of “motherhood”, impacting their subsequent child-rearing and interactions with their own children. Because many comfort women survivors frequently lived in abusive and exploitative adoptive families as adopted daughters/daughters-in-law, these early-life experiences provide an important context for understanding the complexity of their experiences of motherhood.

### Mothering and family life in the aftermath of conflicted-related sexual violence

Research from the field of CRSV and war-related experiences suggests that a history of trauma has been shown to place people at risk for the persistence of psychological and mental distress such as depression, anxiety disorders, and PTSD (Kellermann, 2001a; Kuwert et al., 2014; Schnurr et al., 2003). One study on 20 aging Korean comfort women survivors showed a high rate of current (65%) and lifetime (90%) PTSD (J. Lee et al., 2018). Moreover, research in the context of the Holocaust (Kellermann, 2001b), war-related sexual violence (Roupetz et al., 2023), interpersonal violence, and childhood maltreatment (Bailey et al., 2012; Banyard et al., 2003) has highlighted that trauma can affect not only the primary trauma survivor, but also subsequent generations in complex ways, including parent–child relationships (Bachem et al., 2021; Kellermann, 2001b). Furthermore, research has shown that PTSD is related to increased levels of parenting stress, less optimal parent–child relationships, and more frequent use of negative parenting practices, such as overt hostility and controlling behavior (Christie et al., 2019). In turn, these can negatively impact the health, development, and attachment security of subsequent generations (Lambert et al., 2014; Udita et al., 2014; Wachs et al., 2009).

These interfamilial and intergenerational realities highlight the possibility that in the aftermath of CRSV, survivors’ mothering and family life may be both complicated and challenging. Most research on the motherhood experiences of CRSV survivors has focused on women with children born of CRSV. For example, mothers of children born of CRSV bear the burden of being excluded by their communities; survivors and their children often face high levels of stigma, and the children can be at risk of abuse, abandonment, and marginalization (Denov et al., 2018; Woolner et al., 2019). Studies on women who bore children as a result of wartime rape have revealed that mothers and their children often experienced ambivalent relationships. Here, the children often served as powerful reminders for mothers of their suffering from the rape (Denov et al., 2018; Kahn & Denov, 2022; Mukamana & Brysiewicz, 2008; Woolner et al., 2019). Nevertheless, despite the challenges of raising children born of CRSV, motherhood can also be a source of strength for many survivors of CRSV. In many cases, motherhood was perceived by survivors of rape as a positive and meaningful resource to overcome the effects of genocidal rape, fostered positive emotion, provided a reason to live, and to imagine and shape possibilities for life post genocide (Kantengwa, 2014; Zraly et al., 2013).

This literature review has highlighted the socio-historical, cultural, familial contexts, and patriarchal norms and constructions of gender and motherhood in Taiwan and beyond, particularly in the aftermath of war and CRSV. These contexts and norms frame the lives and realities of the families of comfort women survivors, who are the focus of this study.

## Methodology

This qualitative study was embedded within a larger research study, conducted between November 2021 and June 2022, exploring the impact of early-life conflict-related sexual trauma on Taiwanese comfort women survivors and how family members perceived survivors’ experiences of sexual victimization. The findings for this analysis are based on in-depth interviews in Taiwan with the families of three deceased Taiwanese Han-Chinese comfort women survivors, examining family members’ perspectives on survivors’ mothering, family life, and intergenerational realities.

We used a narrative inquiry approach to our analysis, which draws on stories where “events and happenings are configured into a temporal unity by means of a plot” (Polkinghorne, 1995, p. 5) to describe the complexity of human actions. Narrative inquiry is particularly relevant for this study because it facilitates the exploration of participants’ lives, while reflecting on the context in which their narratives are embedded (Clandinin & Connelly, 2004). It also facilitates a focus on a smaller number of individuals, while gaining a deeply nuanced understanding of the narrators’ life experiences (Creswell & Poth, 2018). We collected stories of personal experiences through the lens of the three-dimensional narrative space (Clandinin & Connelly, 2004), attending to temporality, place/situation, and social interaction of the events and experiences participants described. This approach can illustrate and illuminate the connections between individual narratives and the systems of domination that impinge upon the everyday lives of comfort women survivors and their families, as well as the intergenerational effects.

### Recruitment

At the time of recruitment, only one comfort woman survivor was known to be living in Taiwan. Given the limited number of surviving comfort women in Taiwan, it was decided to interview deceased survivors’ families to understand more about their experiences and perceptions of family life. In total, three Han-Chinese families, consisting of eight family members including both second and third generations, were recruited through those associated with the survivor redress movement for the current study. The second generation included four females ranging in age from 60 to 74 years, comprising three daughters and one daughter-in-law of the survivors. The third generation represented the survivors’ grandchildren, including three men and one woman ranging in age from 36 to 49 years.

### Ethical considerations

This study received approval from the Research Ethics Board of McGill University. All participants provided written or oral informed consent prior to participating. Participants were informed of the purpose of the research, the voluntariness and anonymity of their participation, and their withdrawal rights. They were also informed that in case of any emotional discomfort caused by the interview process, they would be provided a referral to an appropriate resource to discuss their emotional reactions.

### Interview procedure

The interview protocol included questions regarding what participants knew about their mother/grandmother's life story, how sexual trauma may have affected their mother/grandmother's gendered roles (e.g., daughter, wife, mother), and in what ways had their mother's/grandmother's life story affected their own lives. Interviews were conducted by the first author, who speaks fluent Mandarin Chinese. Seven participants’ first interviews were in-person, while one requested a telephone interview. After the first author transcribed the participant's first interview, if there was additional information needing exploration, a second interview was conducted. Two participants were interviewed once, and six participants were interviewed twice, which amounted to 12 interviews. Participants were interviewed individually or in a group from the same family if the participant requested to be interviewed with his/her family member. Each interview lasted between 90 and 180 minutes. The interviews were digitally recorded and later transcribed for coding and analysis.

### Data analysis and presentation of the data

We used holistic-content analysis (Lieblich et al., 1998) to facilitate a holistic understanding of the data. The raw data were analyzed in Chinese by the first author to ensure that the original meaning was not lost during translation. The data analysis began with reading interviews numerous times, followed by line-by-line coding. The global impression of the three families were documented and the transcripts were color-coded according to unfolding events, epiphanies, and turning points in participants’ accounts. Then, both authors engaged in many discussions, through in-person and Zoom meetings related to the analysis, to discuss the interpretation of the narratives, and themes generated from the stories to contribute to the study's trustworthiness. A collection of quotations representing the themes or particularly significant findings were selected from the transcripts and translated into English for data presentation. The translation was proofed by a Taiwanese fluent in both languages to ensure the trustworthiness of the translation.

To present the findings, we used composite narratives, which synthesized research participants’ accounts to present them as a single narrative (Taber, 2013; Willis, 2019). Composite narratives are used to protect the anonymity of research participants including public figures (Willis, 2018) and vulnerable populations (Sikes & Piper, 2010). The use of composite narratives is relevant for this study, because some of the deceased survivors discussed in this study were involved in the comfort women redress movement and their stories were widely publicized in Taiwan and internationally. As such, simply using pseudonyms or altering survivors’ personal characteristics could still identify family members. We chose composite narratives, which masks individual stories, as a vigorous approach to protect the participants’ anonymity and confidentiality. We applied the approach Willis (2019) proposed using third-person narratives with composite characters to present data from several interviews as if it were from a single individual. All the quotes in this article are direct verbatim from the original transcripts, and all contents of the composite narratives are from the original interviews, not fictitious ones.

A composite narrative was created combining the three families’ accounts into a single family narrative (Ling's family). This was accomplished using the five-element problem-solving approach of narrative structure: (a) characters, (b) situations, (c) problems, (d) actions, and (e) outcomes, all of which are used as plot elements to weave the storyline (Ollerenshaw & Creswell, 2002). It should be noted that Ling's early-life background has been modified from the oral history of Huang A-tao (Chu, 2009; Lai et al., 2005; TWRF & Hsia, 2005), the first Taiwanese comfort women survivor who publicized her identity, to present the multiple structural oppressions in Han-Chinese survivors’ lives. Ling's daughter Hui is a composite of three second-generation daughters, while Ming and Yu represent the composites of four third-generation participants.

## Findings: Ling's family story—Hui, Ming, and Yu

Ling is a deceased Taiwanese comfort women survivor. Her adopted daughter, Hui, alongside her two grandsons Ming and Yu, shared their family story. At the time of the interviews, Hui was in her early 70s, while Ming and Yu were in their 40s. They elucidated how Ling's motherhood was shaped by sexual trauma and cultural gender norms that further impacted the lives of second and third generations.

### Multiple structural oppressions leading to Ling's comfort woman victimization

As a girl with many siblings in a poor family, Ling was adopted out in childhood. Given the adoptive family's maltreatment and abuse, her birth father brought her back home at age 11. Ling received one year of schooling and quit school when she was 13 because of economic hardship and her parents’ opinions that education was unnecessary for girls destined for marriage, despite the fact that Ling enjoyed school. As a girl, Ling stayed home caring for her siblings and doing chores. She later worked as a maid to support herself and her family. At the age of 20, Ling went overseas to what she thought was a nursing job. However, Ling was deceived and instead was enslaved in a comfort station in Indonesia where she suffered from repeated sexual violence and the brutalities of warfare.

The comfort station was under the Japanese military's surveillance. The manager gave Ling the Japanese name “Setsuko” and she was forced to have sexual intercourse with 20 soldiers from morning until evening. Enslaved for more than a year, Ling contracted malaria, had an appendicitis, and her right eye was blinded by shrapnel. She once considered escaping from the comfort station but was caught by a Japanese military policeman. She contemplated suicide several times but later gave up the idea in the hope she could survive and return to Taiwan to see her family. In 1945, Japan was defeated and with the support of a Taiwanese association in Indonesia, Ling returned to Taiwan.

### Ling's post-war silence and shame

After returning home, Ling never revealed her comfort woman experience to her family. Ling did not expect to get married. However, in her late 30s, through a friend's introduction, she married a retired soldier from China while hiding her sexual trauma from him. The couple adopted two girls because of Ling's infertility from her comfort woman experience.

Ling never revealed her comfort women's experience to her family until her participation in the comfort women's redress movement in the 1990s, when she was in her 70s. During interviews, Ling's family made links between her sense of inferiority, her comfort women experience, and her infertility. Ling's grandson Ming expressed that his grandmother's strong sense of inferiority prompted her to worry that her family would look down upon her. As Ming explained, this worry was not in vein:

The only people who knew she had experienced [comfort women system] was my grandma's sister … When quarreling, she sometimes used this to provoke my grandma, “Ah, you were a prostitute!”

Ming explained that because of shame and her fear of insults, his grandmother would invoke a domineering presence with others:

She might be more aggressive and even oppressive when communicating with others. But when others were not present, she was actually terrified … She was scared of being gossiped about. No matter what kind of gossip, she could get mad at trivial things …

Ming's younger brother Yu's accounts of Ling's marital relationship served as a backdrop to understanding Ling's struggles. Although Ling's husband reportedly provided essential economic family support, he had a long-term affair. Although aware of the affair, Ling focused diligently on caring for her family. Yu thoughtfully recounted what life was like for his grandmother, linking her choices and ongoing pain to her experience as a comfort woman. Yu described how he, as a child, became implicated in the story:

My grandpa was married on the Mainland [before 1949]…. He was forced to come here [Taiwan]. At that time, it was not allowed to travel [to the Mainland] to visit relatives. So, he re-married [my grandma]^1^. However, my grandma could not give birth, so grandma felt guilty … this is also one of the tangled issues in her heart … So, when this man went out to do such a thing [extramarital affair], she would carry me to the mistress's house and [my grandmother] could only stand outside watching them. After looking for a while, she just went home and didn’t dare to do anything … I wonder how she could painfully endure this. She never told anyone [about her comfort women experience], and no one in her family knew; how did she hold on? In addition, she didn’t dare to say a word about her husband's betrayal. Was it because of her sense of inferiority …?

### Motherhood and intergenerational realities: Navigating sexual trauma and cultural gender norms

Ling's experiences of motherhood appeared to be shaped by the interplay of sexual trauma and traditional Han-Chinese cultural gender norms—particularly, the necessity for a woman to have male offspring for family inheritance. As Ming described, the destruction of her reproductive health because of her comfort women's experience brought Ling significant trauma:

She thought a mother is the core and pillar of the whole family. So, to make her life complete, she should have a child to care for and not let people doubt her personality or abilities because of it [infertility] … She was a person who cared about other people's perceptions, so when family, the most significant part of her life, was deprived from her, I think the trauma inflicted on her was huge.

Ling adopted Hui for the purpose of family inheritance. When Hui grew up, Ling requested that Hui have an uxorilocal marriage. Hui would thus remain with her adoptive family under the condition that her first son must carry the adoptive family's name. Hui later gave birth to three male children (Ming, Yu, and their older brother) and one daughter. As promised, Hui had a first male child to inherit the family name, forging intergenerational ties.

According to her family, Ling's comfort woman trauma impacted the second and third generations, through ambivalent mother–daughter relationships, conflictual relations because of male preferential treatment, and the intergenerational impacts on marriage and parenting.

#### Ambivalent mother–daughter relationships

Hui reported an ambivalent, conflictual, yet simultaneously affectionate and sympathetic, mother–daughter relationship with her mother Ling. Hui explained how she perceived her mother's experiences of sexual victimization to have impacted their relationship. Ling was reportedly unable to establish trusting relationships with others, including her two adopted daughters. Hui's older sister reported leaving home early because of what she perceived as Ling's “over-controlling mothering”, while Hui stayed with Ling in an uxorilocal marriage that Ling herself had arranged. Hui explained why she could not just leave home, “I can’t be cruel. I would worry that no one will take care of them [my parents].” At a young age, Hui recalled that she was compelled to be obedient and take care of the family chores:

… As a 13-year-old child, I don’t know how I learned how to cook … I purchased meat … and two or three kinds of vegetables, cooked and ate at noon, and went to evening classes after three o’clock. Sometimes I raised chickens and ducks; I raised all of them by myself … She did her things, and I made lunch [a wry smile]. I was pretty dutiful to my role …

Ling's controlling mothering style reportedly influenced Hui's life profoundly, including her social network, marriage, and psychological well-being. Hui expressed the long-term tense mental pressure as a child that led to her later depression:

I was very timid, afraid of doing things wrong, and fearful of being beaten or scolded for it … Every morning when I got up, I would gauge what kind of mood she was in, and be compliant. Do you see how nervous I was? I was depressed for a while … I told myself I needed to stand up. If I continued to be depressed, this family would fall apart.

Ming highlighted the ways in which his mother Hui still lived in his grandmother's shadow of emotional abuse caused by Ling's past trauma, even if Ling was no longer living:

Although grandma is gone, my mother is still here. However, the influences left by my grandmother still bore on my mother's emotional state, why does my mother have to bear these things? … My mother would feel that everything was her fault. Isn’t that being the same as my grandma?

Despite the long-term tense family relationships, Hui ultimately respected her mother's devotion to the family and was highly sympathetic to her mother's complex life course. She described her mother as the person to hold the family from falling apart:

She held the family together; she didn’t let it break apart. She was also hardworking. Without her, this family would disperse. She was very strict with us, but she was a good mother. She had fulfilled her responsibility as a mother. So, I would be considerate of her position. Because her husband would not give her peace of mind, she had to fight on her own [to raise this family]. It was very hard. It was not easy for a woman to sustain a family like this.

#### Conflictual relationships resulting from the interplay of sexual trauma, gender norms and male preferential treatment

Hui described her ongoing conflict with Ling, which stemmed from Ling's unequal affection toward her four grandchildren. Ling favored the one carrying the family name, as she had no sons of her own due to infertility and relied on this first grandson to carry on the family legacy. Hui described that she was often caught between Ling and her children, creating conflictual relationships. “When I was teaching the children, grandma would add fuel by the side. Then when I didn’t discipline them, she would scold me, saying that I didn’t know how to discipline children.” Yu, the grandson concurred, indicating that his grandmother's over-indulgence of his eldest brother led to his brother's misbehaviors and created family tensions:

My brother behaved badly … If he wanted money, grandma would give him money. If he misbehaved, she would put the blame on my mother. She would say, “Why don’t you discipline this child?”.

Hui noted that her mother, being influenced by cultural traditions, considered her eldest grandson to be the person to worship her after she died and, therefore, doted on him. Hui described an incident that exemplified Ling's preferential treatment and family conflict:

He [the eldest son] was in army service and came back on leave. My mother made chicken stew for him … It just happened that Yu came back from work, but she didn’t mention that she had cooked stewed chicken, and did not let the brothers eat together; she didn’t say anything. I was at home just that day, and I said [angrily] “…You should give Yu a bowl [of chicken stew] … Are you fair? Isn't he born from me? … Otherwise, why don’t you let him eat?” At that time, I was young, and the way I talked was very rough and rude.

#### Marriage and (grand)parenting

According to family members, the interplay of Ling's sexual trauma and cultural gender norms also appeared to impact the marriages of the second and third generations. Hui described the events leading up to her own uxorilocal marriage arranged by Ling: “This [my marriage] was what they [my parents] arranged. Not by my will. I got married just for the sake of getting married …”. Hui was torn between her husband and Ling because of the conflicted relationship between the two. As a result, Hui divorced her husband after a few years of marriage, never remarrying because she wanted to avoid future intra-familial suffering:

One was my husband, and the other was my mother; what should I say? … I won’t get married again … If a man has to live under the rule of his wife's family, he would become scorned because of me … so why bother? I could bear it alone; I didn’t want anyone to suffer like me.

Despite the conflicts between their mother and grandmother, both Ming and Yu reported their grandmother's deep love for them and their appreciation for their grandmother. Ming pointed out that being a grandmother was a “redemption” for his survivor grandmother, who suffered profoundly from her past sexual trauma:

If someone wants to help her, if it were me, I would do it this way: I would tell her [my grandma] you are wonderful, you know how to be compassionate, you also know how to love, and you don’t have an inferiority complex, so there is no wall between you and your grandchildren. So, just interact with other people in the same way.

Nevertheless, despite there being no direct tension between themselves and their grandmother, Ming and Yu reported that they felt their personalities had been influenced by their grandmother, the efforts to protect their mother from their grandmother, their spousal choices, and perspectives on marriage. Ming expressed that to protect his mother from his grandmother's volatile temper, he followed his grandmother's expectation to study hard:

… We didn’t want her [my mom] to have such a hard time every day. So, we tried not to make grandma angry; and tried to live up to grandma's expectations … She expected me to study hard. Therefore, I studied hard so she wouldn't get angry …

Ming further stated that his grandmother had a “reverse influence” and that he sought to engage in different behavior from what his grandmother would do. Ming strongly identified with his mother, choosing a spouse similar to his mother, *“*Unconsciously, I chose a person whose personality is more similar to my mother's … Basically, [a person] being dominant will not be a good match for me”. Similarly, Yu described how witnessing Ling's struggle in her marriage strengthened his belief that he needed to protect his wife and his family, “I don’t want grandpa's… shadow … having a mistress outside [of marriage]*.*”

Addressing how Ling's trauma may have influenced the parenting of second and third generations, Hui reported how she felt that her relationship with her children was affected: “I felt that I restricted them [my children] too much … I have been gradually adjusting myself, stopping interfering with what young people want to do …”. Ming noted that Hui supported him and his siblings: “She [my mother] is somewhat influenced by grandma, so she doesn’t want to be like grandma. She wants to be a mother who is more willing to relate to children.”

Yu, Ling's younger grandson, was raised by Ling when Hui was busy with work to support her four children, as well as Ling. Yu described Ling's preferential treatment of his elder brother and her inattention toward him, which made him feel aggrieved. He noted that when he was young, he wondered why Ling favored his eldest brother:

“Is it because he is your inheritance?”*…* It was only after I grew up that I realized she was influenced by the concept of the bloodline *…* She did not mean to be so good to my brother and treated me as inferior*.*

Now, being a father of two children, Yu noted his responsibility as a parent, “When I married and had a child, I knew that raising a child is not easy, and you must educate him.” He was aware that he was struggling with his parenting, he worried he would replicate Ling's parenting, “I’ve always told myself that I can’t repeat the same mistake. Like my grandma's situation, the indoctrination [of materialism] on children. But until now, I haven’t been able to break through this point ….”

### Walking out of the shadows: Change, activism, and resilience

Although Hui, Ming, and Yu were deeply affected by Ling's sexual trauma, they demonstrated their longing and capacity for change and resilience. Participants’ attempts to “walk out of the shadows” of conflict-related sexual violence was revealed in their efforts to “break the family curse”, and their participation in the comfort women redress movement.

#### Breaking the “curse”: Efforts by the second and third generations

Hui, Ming, and Yu recalled that in the latter period of Ling's life, the conflictual family relationships intensified, yet eased when Ling passed. Ming noted,

After grandma passed away, we lost something, but also gained something … She [my mom] is still adjusting to her new life, and although she still has some shadows from her childhood, she is moving toward a new positive direction.

Hui discussed Ling's legacy and the importance of halting the intergenerational transmission of negative influences: “I don’t want to transfer the stress I’ve suffered to my children, so I try to keep my children out of the way as much as possible*.*” From Ming's perspective, his mother Hui has been very supportive. Ming completed his college education and had been working diligently to support the family: “I wanted to grow up and make money for my mother so she would not need to work so hard.” He also noted what he perceived he had gained from the tense family dynamics: “… People say that children in adversity would grow stronger. That's it. I won’t let my family worry about me.” The grandchildren saw their family's business venture as an effort to break the family “curse” of being conservative and risk-averse:

Because of my grandmother, my mother always behaved cautiously, and was extra-careful to avoid anything that could upset the peace. Her behavior had a significant impact on me … I later became the kind of person who refused to take risks because you didn’t know when grandma would lose her temper again….

I think for our whole family starting a business was like a collective decision to escape from the framework [of daring to risk]. Yes, that feeling was like “breaking the curse.” As I said, our family is so conservative. No one would dare start a business … But when there was an opportunity, when there was just a spark of opportunity, unexpectedly, everyone supported it and participated … everyone collectively wanted to break out of the old framework.

#### Participation in the comfort women redress movement

With her family's support, Ling participated in the comfort women's redress movement for 20 years, enriching her later life. As Ming noted, “When she entered the Foundation [TWRF],^2^ I gradually felt that they drew out her memories, not only the bad ones but also the wish for the happiness she had longed for.” Hui added that “She [my mother] said everyone [in TWRF] would call her ‘Grandma’. She was pleased.” Her family highlighted how TWRF healing activities supported their (grand)mother's recovery:

She attended some [healing] courses, and the guidance teacher [counselor] would counsel her. When she returned home, she would tell us what courses she went to and what the teacher taught her … So, we realized that there was a feeling of relief [from the past regrets] in her mind …

Demonstrating their own activism, her family participated in the redress movement: “I accompanied grandma [my mother] … [because] she said, ‘if you can be my company, I would be feeling more courageous’”. Ming felt gratified to contribute to the comfort woman issue. He helped organize his grandmother's possessions after her death to honor and memorialize her struggles:

Because grandma has passed on now, there is no one to ask [about their stories for advocacy]. Therefore, I dug up some photos and other things that grandma did not make public … Fortunately, I have participated in this clearing her things. So, those willing to continue discussing this topic in the future can have a context [of the issue] or a record for reference.

Ling's family further expressed that the government's and TWRF's support helped to relieve their financial burden:

My family has been in economic hardship … we received much support from the society.” “I felt relieved [by the government's monthly stipend for Ling]. It was also a relief for my mom. We didn’t need to worry about her [living and medical] expenses.

Through this composite narrative, the complexities of motherhood, parenting, and the intergenerational impacts of the comfort woman system are revealed. Ling bravely bore multiple lifelong traumas from war, sexual violence, and a patriarchal culture that shaped her experiences. Hui endured lifelong stress and hardships that appeared to emerge from Ling's suffering. Nevertheless, Hui's empathy for her mother, support for and from her children, and her self-awareness enabled the family to walk toward a new life chapter, out of the shadows of CRSV. The third generation could not undo the intergenerational relationships influenced by Ling's life experiences. However, empathy, understanding, and deep love for Ling and Hui filled their profound accounts.

## Discussion

Ling's family represented the experiences and narratives of eight family members, including the second and third generations from the family of three deceased Chinese-Han comfort women survivors. Their experiences reflected the deep intertwining of the comfort women experience and structural factors, alongside the ways in which family members—although not direct victims—lived in the shadow of the comfort woman experience. Despite those challenges, the family demonstrated their agency, walking out of those shadows, embodying strength and resilience.

Ling's comfort women's experiences appeared to interplay with survivors’ sexual trauma, reproductive health, and gendered structural pathways, and oppression. Participants reported that this interplay led to long-term psychological distress, shame, and sense of inferiority, further impacting her interpersonal, spousal, and family relationships. Under these constraints, Ling married a retired soldier from China, adopted children to enable motherhood and actively arranged an uxorilocal marriage for her adopted daughter to ensure their family legacies and establish intergenerational ties. Chiang (1996) noted that many comfort women survivors remained single, stayed in cohabitating relationships, or chose to marry widowers or those who had already divorced. These are traditionally considered “deviant” life paths or marriages of lower social status and not the “best” choices for women. Ling's experiences of marriage, motherhood and intergenerational family ties highlight comfort women survivors’ struggle within the intersection of sexual victimization and the structural constraints embedded in their lives, while demonstrating her “agency within structure” (Settersten & Gannon, 2005, p.36) and her social navigation skills and autonomy to construct their life course (Elder, 1994).

Ling's mothering impacted the lives of her offspring. First, the ambivalent mother–daughter relationship between Ling and her daughter Hui appeared to be influenced by Ling's sexual trauma and psychological distress, ultimately impacting Hui's marriage, social network, and psychological well-being. Moreover, Ling's preferential treatment of male offspring alongside her sexual trauma and cultural gender norms was reported to contribute to family tensions, leading to conflictual relationships between Ling, her daughter, and the grandson who inherited the family name. While Hui reported long-term conflictual relationships with her mother, she also described deep affection, sympathy, and respect toward her survivor mother, and respected Ling's dedication to her family. Our study (Kang & Denov, submitted for publication) highlights that after learning about Taiwanese comfort women survivors’ sexual victimization, most family members were empathetic to the effects of early trauma on survivors, took on new ways of seeing the survivors, challenging their perceptions of their grandmothers, ultimately garnering a richer, more complete portrait of the survivors. These simultaneous realities of struggles and compassion characterize their ambivalent relationship in the shadow of sexual violence.

The third-generation participants Ming and Yu reported being affected by family dynamics influenced by Ling's comfort women's experience, particularly through (grand)parenting. Ming and Yu reported that their spousal choices, attitudes regarding marriage, and personality were affected by conflictual family relationships. Ming reported using strategies to mitigate his family conflicts, including behaving well to protect his mother, and identifying with his abused mother. Ming's reactions appeared to be similar to children witnessing family violence, worrying about their parent's well-being (Graham-Berman, 1996) and use opposing strategies to cope with family conflicts (Allen et al., 2003; O’Brien et al., 1991). Moreover, Ling's parenting styles appeared to influence those of her daughter and grandson, which is consistent with research findings that parenting styles are often intergenerationally transmitted (Serbin & Karp, 2003; van Ijzendoorn, 1992).

In contrast to the ambivalent mother–daughter relationship of the second generation, Ling's relationship with her grandchildren was reportedly caring. Grandparenting was a significant channel for Ling to express her love, especially since she had encountered difficulties in interpersonal and family relationships due to profound sexual trauma. This finding is consistent with studies in Chinese cultural contexts where grandparenting is associated with better mental health status (Guo et al., 2008; Tsai et al., 2013; H. Xu, 2019) and life satisfaction (L. Xu et al., 2012), especially in multigenerational households in which adult children were present (Ku et al., 2013).

Ling's family demonstrated their desire and capacity to walk out of the shadows of Ling's sexual trauma. Hui was determined to stop the intergenerational impacts by being a supportive mother, while her grandsons strove to challenge the conservative family culture influenced by conflictual family dynamics. In addition, Ling's family reported that the supportive psychosocial activities provided by non-governmental organizations (NGOs) allowed Ling to heal, consistent with Hung (2010) that therapeutic activities assisted Taiwanese comfort women survivors in reconciling their traumatic experiences. Moreover, the government's support helped to relieve the economic burdens of Ling's family, which was under an intergenerationally accumulated economic-disadvantaged status, illustrating the importance of government policies or support from NGOs for CRSV survivors’ families.

## Conclusion and implications

To our knowledge, this is the first study to explore motherhood and intergenerational implications of comfort women survivors’ and their families including second and third generations. The study has important implications for policy, intervention, and research with comfort women and the families of CRSV survivors. First, including survivors’ families in assessing service needs is essential. Over the past three decades, activists, NGOs, and governments, like Taiwan and South Korea, have sought to achieve legal justice against the Japanese government, raise public awareness, and support comfort women survivors’ economic and psychological distress. These important efforts helped to restore dignity for many survivors whose families and societies disparaged them, yet the impact on survivors’ families has remained insufficient. Examining the family narratives shows how comfort women experiences can have intergenerational consequences. The lives of the second generation, now aged 60 to 80 years, were directly impacted by their mothers’ sexual trauma, requiring attention and support, while the third generation also requires resources to cope with the legacies left by the negative interaction between survivors and their parents.

Second, our study highlights that culture, gender, and family norms regarding women's life paths, and sexuality play an important factor shaping survivors’ psychological well-being, motherhood and family lives. Addressing vital cultural factors in the intervention with comfort women and other CRSV survivors and families is essential. This is consistent with research (Denov & Piolanti, 2019) advocating the importance of including a culturally appropriate family approach to address the shared trauma, ambivalence, and challenges in the mother–child relationships when assisting CRSV survivors and their children born of rape. Our findings indicate the positive effects of healing activities, social activism, and the need for a culture-sensitive family approach to assist survivors of CRSV and their families. A comprehensive service model for comfort women and their families requires a culture-driven multilayered approach at individual, familial, community, and societal levels.

At the individual level, the provision of psychological and social support services (such as counseling/therapy, support groups, and comprehensive case management), together with the adoption of suitable government policies (such as economic and medical support), can address individual survivors’ and family members’ psychosocial needs. At the familial level, family counseling/therapy on trauma recovery, mother–children/intergenerational relationships, and parenting issues would foster positive mother–child relationships, among others. Finally, at the community and societal levels, it is vital to develop campaigns, social education, and policy reforms addressing the issue of violence against women that duly consider the relevant gender norms and structural factors. All these macro-level efforts can help CRSV survivors and their families move past the harms of sexual violence and encourage them to assert their rights in the present and future, and to continue to walk out of the stigma and shadows of CRSV. Moreover, to assist survivors and their families effectively, it is important for helping professionals be cognizant of the complex interplay of history, culture, structural and intersectional factors and the ways in which they shape survivors and their families.

In terms of research limitations, no comfort women survivors, and no second-generation men participated in this study, rendering their perspectives unavailable. Despite these limitations, the in-depth information revealed by participants provided an intergenerational view of the families of comfort women survivors. Further research of families of comfort women survivors can help to support the design of a comprehensive service model to other CRSV survivors and their families. Also, the second and third generations should be consulted about their service and policy needs.

Comfort women remain a profound stain and shame in most Asian victimized countries, with survivors not revealing their victimization to their families or having experienced rejection if they disclosed their past. These factors increase the challenges in reaching out to survivors’ families regarding research in Taiwan and South Korea (J. Lee & Lee, 2019). Our composite narratives serve as an ethical approach for researchers to engage comfort women's families in research to protect their privacy and anonymity. We also urge the governments in countries affected by the comfort women system to work with and support researchers and activists to increase the feasibility of reaching out to survivors’ families and providing necessary support.

Wartime sexual trauma has lasting effects on individuals and their families. Our intergenerational study of three deceased Taiwanese comfort women survivors’ families highlights the ways in which survivors’ sexual trauma impacted their motherhood and led to long-term effects for their offspring. Our study underscores that the trauma of comfort women's experiences deserves ongoing attention and service provision with a culture-sensitive multilayered intervention frame to mitigate intergenerational impacts and foster resilience.

## Notes

1. In 1949, Kuomintang (KMT, the Nationalist Party), the ruling party of the Republic of China led by Chiang Kai-shek, was defeated by the Communist Party and fled to Taiwan from China. Many male soldiers came to Taiwan with KMT while leaving their spouses, children, and families in China. Given the hostile cross-strait relations between Taiwan and China, people were prohibited from traveling and communicating from both places.

2. Taipei Women's Rescue Foundation (TWRF) is the only non-profit organization dedicated to the comfort women issue in Taiwan. It supported the survivors’ redress movement against the atrocity of the Japanese government and military while providing 16-year-long supportive group activities for survivors’ trauma healing (Kang, 2021).
